# Stroke in a 32-year-old male with hypertension, hyperlipoproteinemia(a), and no large vessel disease: a case report

**DOI:** 10.1093/ehjcr/ytaf309

**Published:** 2025-06-27

**Authors:** Zhenwei Gao, Moe Arai, Hanzhang Ma, Jianyu Chen, Pingsheng Wu

**Affiliations:** East-West Medical Center, Rm 1401, Office Tower, CITIC Plaza, 233 Tianhe North Road, Guangzhou 510613, China; Oka Medical Center, Rm 501, 5th Floor Tower D, GT Land Plaza, No. 8, Zhujiang Road West, Zhujiang New Town, Guangzhou 510627, China; The First Clinical Medical College, Guangzhou Medical University, Guangzhou 511495, China; Department of Neurology, The Second Affiliated Hospital of Guangzhou Medical University, Guangzhou 510260, China; Department of Radiology, Sun Yat-Sen Memorial Hospital, Sun Yat-Sen University, Guangzhou 510120, China; East-West Medical Center, Rm 1401, Office Tower, CITIC Plaza, 233 Tianhe North Road, Guangzhou 510613, China; Oka Medical Center, Rm 501, 5th Floor Tower D, GT Land Plaza, No. 8, Zhujiang Road West, Zhujiang New Town, Guangzhou 510627, China; Department of Cardiology, Nanfang Hospital, Southern Medical University, Guangzhou 510515, China

**Keywords:** Lipoprotein(a), Cerebral microinfarct, Small blood vessels, Case report

## Abstract

**Background:**

Lipoprotein(a) [Lp(a)] is associated with cardiovascular events through three primary mechanisms: promoting atherosclerosis, thrombosis, and inflammation. While evidence of Lp(a)-induced thrombosis in large vessels is limited, its effects on small vessels have not been previously reported.

**Case summary:**

A 32-year-old male with hypertension presented with 2 weeks of right-sided numbness. Magnetic resonance imaging of the brain revealed multiple punctate lesions in the subcortex of both cerebral hemispheres, as well as a subacute ischaemic lesion in the thalamus. Notablely, no significant evidence of vascular sclerosis or stenosis was found. Blood tests indicated elevated Lp(a) levels. Following treatment with a proprotein convertase subtilisin/kexin type 9 (PCSK9) inhibitor and dual antiplatelet therapy, the patient made a full recovery.

**Discussion:**

Multiple punctate lesions in the subcortex of both brain hemispheres are common but highly non-specific. Further research is needed to determine whether these lesions represent a clinical manifestation of microthrombosis induced by elevated serum Lp(a) levels. The subacute ischaemic lesion in the thalamus suggests that the procoagulant effect of Lp(a) may contribute to cardiovascular diseases, including those affecting small vessels. However, the potential impact of hypertension-induced damage cannot be excluded.

**Conclusion:**

This case highlights the need for thorough etiologic evaluation in young stroke patients with unclear risk factors. Markedly elevated Lp(a) may contribute to a prothrombotic state even without large-vessel disease. Incorporating Lp(a) measurement into diagnostic and therapeutic assessments could aid management, with PCSK9 inhibitors and dual antiplatelet therapy offering potential benefit.

Learning points

- Elevated Lp (a) levels may contribute to thrombosis, leading to ischaemic lesions in small vessels.

## Introduction

Lipoprotein(a) [Lp(a)] is a genetically determined lipoprotein that is strongly associated with increased risk of atherosclerotic cardiovascular disease (ASCVD). Approximately 20% of the global population have elevated Lp(a) levels, and several cardiovascular societies now recommend at least one lifetime screening in all adults. Recent studies indicate that the risk of cardiovascular events associated with high Lp(a) may exceed that of elevated low-density lipoprotein cholesterol (LDL-C).^1^ Although up to 30% of individuals of African descent exhibit markedly elevated Lp(a) levels (>500 mg/L)—the highest globally—their ASCVD incidence is not proportionally elevated, suggesting that the phenotypic expression of Lp(a)-related risk may depend on coexisting vascular or inflammatory factors.^2^

Lp(a) is thought to exert pathogenic effects through three main pathways: promoting atherosclerosis, enhancing thrombogenesis, and inducing vascular inflammation. While its contribution to large-vessel coronary and carotid artery disease is well established, its role in small-vessel occlusive stroke remains poorly defined and largely unreported.^3^ This case explores the potential contribution of markedly elevated Lp(a) in a young male with no diabetes, no arrhythmias, and no major atherosclerotic burden—highlighting the diagnostic and therapeutic challenges posed by an unusual vascular phenotype, and exploring the potential contributory role of elevated Lp(a).

## Summary figure

The first MRI scan, performed on 19 April 2023, revealed a left cerebral infarction in the temporal lobe. The imaging included (A) T2WI, (B) T1WI, and (C) T2WI-FLAIR-Cor. Notably, diffusion-weighted imaging (DWI) was not conducted during this scan. The second MRI scan, conducted on 16 September 2023, revealed ischaemic lesions in the left thalamus in addition to the previously identified left cerebral infarction in the temporal lobe. The findings included: (D) T2WI showing a slightly high signal, (E) T1WI showing an equal signal, (F) T2WI-FLAIR showing a slightly high signal, (G) DWI showing a slightly high signal, and (H) apparent diffusion coefficient (ADC) showing an equal signal. The lack of a significant decrease in the ADC signal suggests that the cerebral infarction is in the subacute phase (3 days to 2 weeks).

**Figure:**
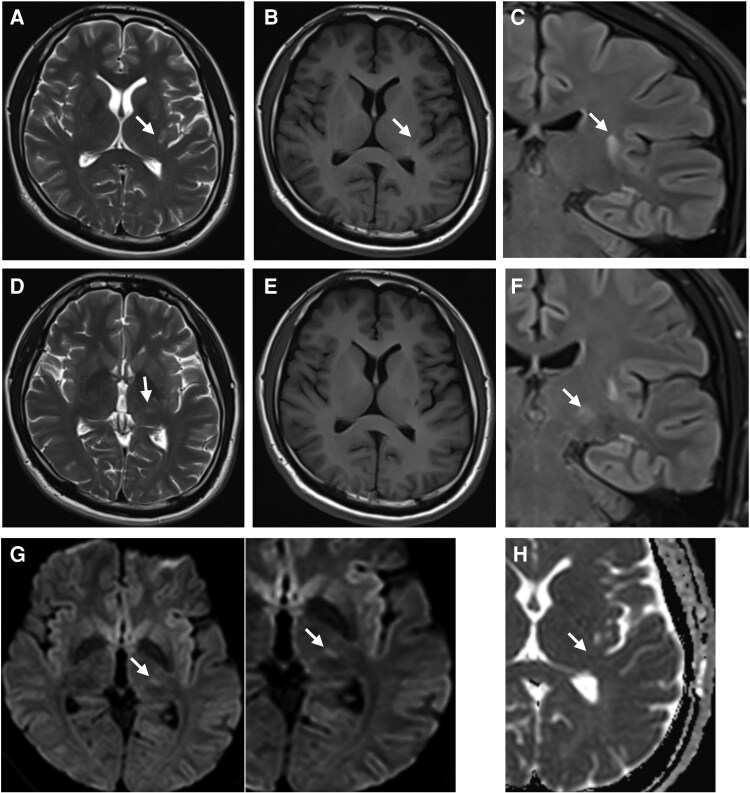


## Case presentation

The patient presented to our clinic on 16 September 2023, with a 2-week history of right-sided numbness. Initially, he reported intermittent numbness in his right upper and lower limbs, which spontaneously resolved within minutes. However, the symptoms began to recur with increasing frequency and duration, ultimately becoming persistent and affecting the right arm, leg, body, and face. He denied any muscle weakness, loss of consciousness, or speech difficulties. Vital signs on admission were as follows: blood pressure 149/106 mmHg, heart rate 75 bpm, height 170 cm, weight 75 kg (BMI: 26.0 kg/m²). Neurological examination showed reduced sensation in the proximal right arm, while other sensory responses were normal, and Babinski's sign was negative.

He had no history of prior stroke, transient ischaemic attack, or neurological symptoms. His past medical history was otherwise unremarkable: he had no known diabetes, dyslipidemia, or arrhythmias, and his fasting glucose and HbA1c levels were normal. He denied alcohol or illicit drug use, but reported a 10-year smoking history, which he had ceased shortly before presentation. There was no personal or family history of stroke or myocardial infarction, although both his father and grandfather had longstanding hypertension. In April 2023, shortly after the patient was diagnosed with severe hypertension at a young age and without other apparent cardiovascular risk factors, an outpatient brain MRI was ordered as part of the initial workup to evaluate for possible hypertension-related end-organ damage. At that time, the patient reported no neurological symptoms. He had blood pressure of 140/100 mmHg noted 10 years ago but did not undergo further evaluation or treatment for secondary hypertension at that time. A secondary hypertension workup was considered during the recent evaluation, but the patient initially declined further testing. He was a smoker, and both his father and grandfather had a history of hypertension. In April 2023, he was formally diagnosed with hypertension (182/137 mmHg) and initiated on antihypertensive treatment with olmesartan and amlodipine. Electrocardiography (ECG), carotid ultrasound, and fundus photography showed no abnormalities, and the urine albumin-to-creatinine ratio was 13.93 mg/g. Following treatment, his blood pressure reduced to 123/89 mmHg.

**Figure 1:**
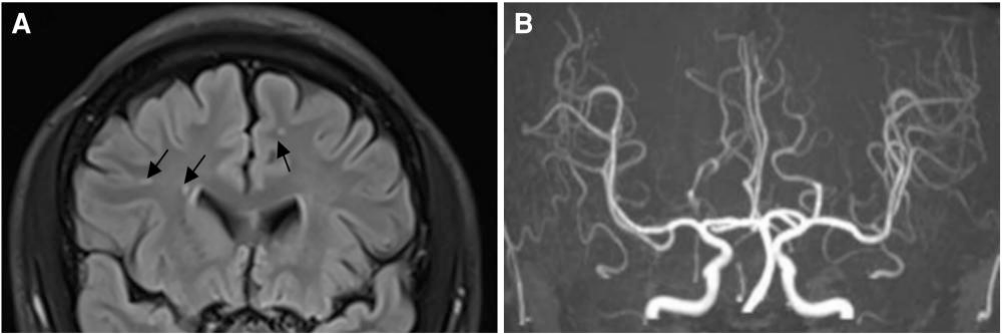
Brain MRI (*A*) and MRA (*B*) before stroke. (*A*) Brain MRI was performed when admitted for hypertension, FLAIR sequence reveals small, scattered high-signal lesions (2–6 mm) in both hemispheres (Arrow), probably due to prior micro infarction. (*B*) Brain MRA was performed when admitted for hypertension, proving normal cerebral arteries without stenosis or occlusion.

**Figure 2:**
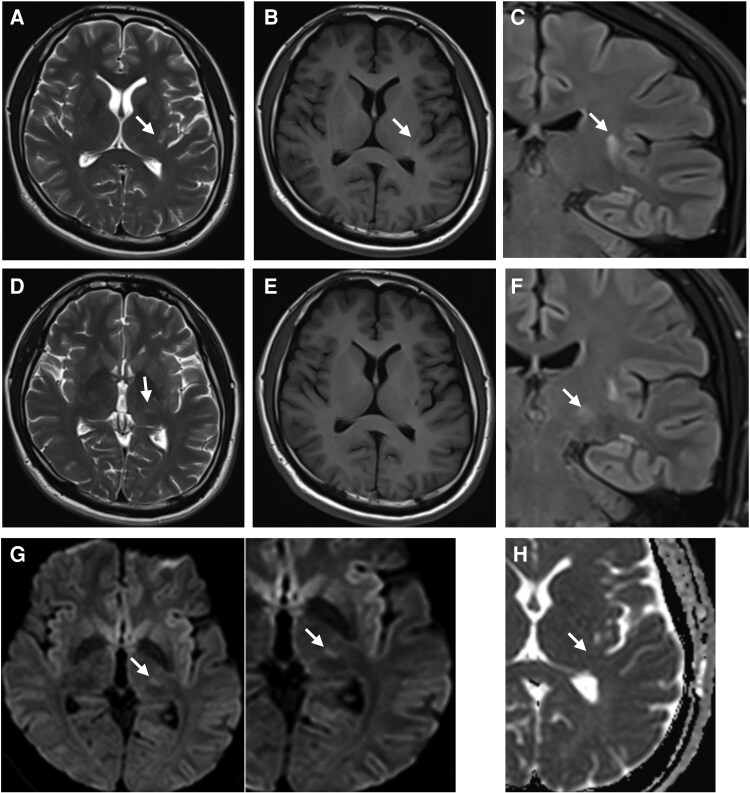
MRI Scans showing progression of cerebral infarction. (*A–C*) The first MRI scan, performed on 19 April 2023, revealed a left cerebral infarction in the temporal lobe. The imaging included (*A*) T2WI, (*B*) T1WI, and (*C*) T2WI-FLAIR-Cor. Notably, diffusion-weighted imaging was not conducted during this scan. (*D–J*) The second MRI scan, conducted on 16 September 2023, revealed ischaemic lesions in the left thalamus in addition to the previously identified left cerebral infarction in the temporal lobe. The findings included: (*D*) T2WI showing a slightly high signal, (*E*) T1WI showing an equal signal, (*F*) T2WI-FLAIR showing a slightly high signal, (*G*) DWI showing a slightly high signal, and (*H*) ADC showing an equal signal. The lack of a significant decrease in the ADC signal suggests that the cerebral infarction is in the subacute phase (3 days to 2 weeks).

Prior to the stroke, the patient underwent a brain of magnetic resonance imaging (MRI, FLAIR sequence) on 19 April 2023, due to hypertension and no other symptoms, which revealed small, scattered high-signal lesions (2–6 mm) in the subcortex of both hemispheres, consistent with Fazekas grade I white matter changes (*Figure 1A*). Additional abnormal signals were noted in the deep white matter of the left temporal lobe, though no symptoms were associated with these findings (*Figure 2A–C*). At that time, no thalamic lesions were observed, and magnetic resonance angiography (MRA) showed no vascular stenosis or occlusion (*Figure 1B*). The patient’s blood pressure was well-controlled at the time of the stroke.

On the day of stroke diagnosis, 16 September 2023, brain MRI (FLAIR sequence) revealed a new lesion in the left thalamus (*Figure 2D–H*), which was not present on the prior scan performed 5 months earlier (*Figure 2A–C*). DWI showed a mildly elevated signal in the thalamic lesion, consistent with a subacute ischaemic stroke and mild oedema (*Figure 2G*). The ADC signal showed no significant decrease (*Figure 2H*), indicating that the cerebral infarction was in the subacute phase (3 days to 2 weeks). MRA findings remained unchanged, with no evidence of large- or medium-vessel stenosis. Lipoprotein(a) was found to be markedly elevated in two independent laboratories, prompting further evaluation for prothrombotic risk. Routine labs including fasting glucose, HbA1c, renal and hepatic panels, thyroid function, and electrolytes were within reference ranges. Additional relevant lab results, including fibrinogen, hsCRP, and homocysteine, are provided below (*Table 1*).

**Table 1:** Blood test results before and after treatment

	Before Treatment	After Treatment	Normal range
Triglyceride(mmol/L)	1.24	0.97	0.00–1.70
LDL-C(mmol/L)	2.93	0.89	Optimal: < 2.6
			Near optimal/above optimal: 2.6–3.3
			Borderline high: 3.4–4.1
			High: 4.1–4.9
			Very high: ≥ 4.9
HDL-C(mmol/L)	1.25	1.07	1.04–1.55
Lp(a)^a^ (mg/L)	901.4^b^	556.7	0–300
	753.1^c^		
FBG(mmol/L)	4.76		3.9∼6.1
HbA1c(%)	5.5		4∼6
Fibrinogen(g/L)	2.42		1.8∼3.5
hs-CRP(mg/L)	0.55		Low risk: < 1.0
			Average risk: 1.0–3.0
			High risk: > 3.0
Homocysteine(μmol/L)	11.49		15.00–20.00

FBG, Fasting blood glucose; HbA1c, Haemoglobin A1C; HDL-C, High-density lipoprotein cholesterol; hs-CRP, High-sensitivity C-reactive protein; LDL-C, Low-density lipoprotein cholesterol; Lp(a), Lipoprotein(a).

^a^The blood level of Lp(a) before treatment was confirmed in two different institutes.

^b^Immunoturbidimetric assay(Beckman).

^c^Immunoturbidimetric assay(Medical system).

Treatment included dual antiplatelet therapy (aspirin 81 mg and clopidogrel 75 mg daily), statin (atorvastatin 10 mg), and PCSK9 inhibitor (evolocumab 140 mg biweekly) for secondary prevention and lipid optimisation. Hypertension was managed with oral olmesartan (40 mg daily) and amlodipine (10 mg daily). The patient’s right-sided numbness gradually improved, and no new neurological symptoms were observed. As of 12 May 2024, his blood pressure was recorded at 126/94 mmHg.

As of 7 January 2025, the patient remained neurologically stable for over 15 months, with complete resolution of symptoms and no recurrent ischaemic events. He has been symptom-free since starting therapy with clopidogrel, aspirin, atorvastatin, evolocumab, olmesartan and amlodipine. However, these medications do not specifically target Lp(a), rather, they address multiple risk factors.

## Discussion

The Emerging Risk Factors Collaboration gathered records involving 126 634 participants in 36 prospective studies with 1.3 million person-years of follow-up and, after making appropriate adjustments for age, sex, lipids, and other conventional risk factors, found that each 3.5-fold increase in the Lp(a) level was associated with a 10% increased risk for ischaemic stroke. Systematic reviews and meta-analyses have demonstrated hyperlipoproteinemia (a) as an independent risk factor for ischaemic stroke. In fact, elevated Lp(a) levels seem to significantly influence the occurrence of ischaemic stroke in adult patients younger than 55 years old. A noteworthy association with the large artery atherosclerosis ischaemic stroke subtype has also been observed. Another Mendelian randomisation analysis confirmed the causal relationship between elevated Lp(a) levels and large artery atherosclerotic stroke, whereas it failed to demonstrate a causal relationship between elevated Lp(a) levels and small vessel stroke, or lacunar stroke.^3^

Hypertensive small-vessel ischaemic stroke is a well-recognized mechanism, typically associated with chronic arteriolar remodelling and lipohyalinosis, particularly in deep brain structures such as the thalamus. Although more common in older individuals, this condition can also occur in younger patients with poorly controlled or long-standing hypertension. In our patient, despite his relatively young age and the absence of significant large- or medium-vessel abnormalities on MRA, hypertension remains the most plausible proximate cause, given the characteristic location of the lesion. Alternative aetiologies were also considered. The patient had no history of illicit drug use, and although he was an active smoker, there was no clinical or family history suggestive of an inherited thrombophilic disorder. Accordingly, no genetic thrombophilia workup (e.g. Factor V Leiden, prothrombin gene mutation, antiphospholipid antibodies) was performed. While markedly elevated Lp(a) may have contributed to a prothrombotic state, its role as a primary cause in small-vessel ischaemic stroke remains uncertain. This is supported by recent evidence from a prospective cohort study in Japan,^4^ which found that elevated Lp(a) levels were significantly associated with major adverse cardiovascular events in patients with atherothrombotic (large-vessel) stroke, but not in those with small-vessel disease, cardioembolism, or cryptogenic stroke. Given the limited ability of MRA to detect small-vessel pathology, hypertensive microangiopathy remains the most consistent explanation based on the clinical and radiographic findings.

Thalamic lesions can cause contralateral limb numbness, typically without significant limb weakness, which aligns with the patient’s symptoms. Initially, symptoms may improve spontaneously, resembling a transient ischaemic attack, but they can progressively worsen, leading to persistent numbness. This phenomenon is likely due to recurrent thrombosis in small blood vessels, as suggested by the patient's DWI and ADC signals on MRI.

Paroxysmal atrial fibrillation: Strokes typically present suddenly with embolic features; however, this patient's symptoms developed gradually. An ECG, including a 24-h Holter monitor, showed sinus rhythm without evidence of atrial fibrillation.

Hyperhomocysteinemia: Normal homocysteine levels (*Table 1*) rule out this condition as a contributing factor.

Coagulation disorders: This case was considered less likely to involve a coagulation disorder, as the patient had no personal or family history suggestive of inherited thrombophilia, and the stroke affected an isolated small-vessel territory rather than a large-vessel or embolic pattern, which is more typically associated with hypercoagulable states.

A pre-stroke MRI revealed multiple punctate lesions in the subcortical and periventricular white matter of both hemispheres, likely chronic ischaemic foci. No atherosclerosis was detected in large vessels, suggesting that the ischaemic lesions were consistent with lacunar infarcts. While hypertension is a major risk factor for lacunar strokes, this patient is relatively young, and prior examinations showed no evidence of large-vessel atherosclerotic disease on imaging, indicating that hypertension alone may not be the primary cause. Elevated Lp(a) levels may inhibit plasminogen activation and interfere with fibrinolysis, promoting thrombosis. The scattered lesions observed in the subcortical area of this young patient may indicate microthrombosis. Further research is needed to determine whether these lesions represent a clinical manifestation of microthrombosis due to elevated Lp(a). Previous studies have shown that plasma Lp(a) apheresis, which reduces Lp(a) levels in patients with refractory angina and Lp(a) levels > 500 mg/L, leads to immediate symptom relief, highlighting the thrombogenic role of Lp(a).^5^

Although hypertension cannot be entirely excluded as a contributing factor to the ischaemic injury, the potential prothrombotic effects of elevated Lp(a) may warrant increased clinical attention. This raises important questions currently unresolved by existing guidelines, particularly regarding the optimal management of antithrombotic therapy for individuals with elevated Lp(a) in both primary and secondary prevention settings. In this case, dual antiplatelet therapy (aspirin and clopidogrel) was initiated promptly at symptom onset in accordance with secondary stroke prevention recommendations and suspicion of a non-cardioembolic mechanism. Importantly, the patient’s blood pressure was stabilized prior to initiation of DAPT to reduce bleeding risk. Although blood pressure was well controlled at the time of the stroke, the exact timing of ischaemic damage in the left temporal lobe remains uncertain given the absence of symptoms. It is also notable that the patient has experienced at least two documented strokes at a relatively young age, underscoring the complexity of his vascular risk profile and the need for individualized risk-benefit assessment in treatment decisions.

Patients with hypertension, elevated Lp(a) levels, and a history of stroke require aggressive lipid-lowering therapy. Statins, which can increase Lp(a) levels by ∼20%,^6^ and are not the first line of treatment (*Table 2*).

**Table 2:** Visual summary of the case

Timeline	Events
Admitted for hypertension	Performed Electrocardiography, carotid ultrasound, fundus photography and urine albumin/creatinine ratio test, without abnormalities. Brain MRI and MRA were also performed. The patient started oral antihypertensive therapy.
2 weeks later	The patient’s blood pressure was well under control.
5 months later	Admitted for 2 weeks of right-sided numbness. The second time Brain MRI revealed a new lesion in the left thalamus. Blood tests showed elevated lipoprotein(a) levels. The patient started a proprotein convertase subtilisin/kexin type 9 (PCSK9) inhibitor and dual antiplatelet therapy.
1 year later	The patient had remained stable, with complete recovery of limb sensation and no recurrence of transient ischaemic attacks.

It has been demonstrated that various interventions can reduce Lp(a) levels, including ezetimibe, niacin, PCSK9 inhibitors, lipoprotein apheresis, fibrates, aspirin, hormone replacement therapy, antisense oligonucleotides, and small interfering RNA therapies.^7^ However, both the American Heart Association's scientific statement^8^ and the European Atherosclerosis Society's consensus statement^9^ emphasize that definitive evidence linking specific pharmacological lowering of Lp(a) to improved cardiovascular outcomes is still lacking. Currently, lipoprotein apheresis remains the most effective clinically available intervention for reducing Lp(a). FDA approval for Lp(a) lowering with lipoprotein apheresis is indicated only for patients with Lp(a) > 60 mg/dL, a criterion met by our patient, though he declined this option.

Emerging therapies such as the antisense oligonucleotide pelacarsen^10^ and small interfering RNA SLN360^11^ are currently undergoing clinical trials and show promise in reducing Lp(a). For this patient, we selected a PCSK9 monoclonal antibody (evolocumab) as adjunctive therapy, given its documented ability to lower Lp(a) levels by ∼20%–30%,^12^ in addition to further reducing LDL-C. Prior to presentation, the patient had already been receiving statin therapy, which had only a modest effect on lipid parameters and did not significantly impact Lp(a) levels. After more than three months of treatment with evolocumab, the patient’s Lp(a) level declined by nearly 40%, possibly reflecting a synergistic effect with antiplatelet therapy. Large-scale trials of PCSK9 inhibitors have shown that patients with elevated baseline Lp(a) derive greater cardiovascular benefit, although these agents are not yet approved specifically for Lp(a) reduction. Niacin, once considered for this indication, is no longer recommended, as it failed to show clinical benefit in two major outcome trials, as noted by the European Atherosclerosis Society. While the impact of Lp(a) reduction on stroke prevention remains uncertain, the use of a PCSK9 inhibitor in this case was based on its dual lipid-lowering properties and its potential role in secondary prevention and risk modification. Current evidence also does not support targeting aspirin use based on Lp(a) levels, and whether aspirin might be beneficial in patients with significantly elevated Lp(a) remains uncertain.^9^

## Conclusion

This case underscores the importance of comprehensive etiologic evaluation in young patients with ischaemic stroke, especially when traditional risk factors are insufficient to explain the event. Although hypertension remains the most likely proximate cause in this patient, the presence of markedly elevated Lp(a) raises the possibility of a contributory prothrombotic role, even in the absence of large-vessel disease. The potential association between elevated Lp(a) and small-vessel ischaemia warrants further investigation. While no therapies are currently approved specifically for Lp(a) lowering, PCSK9 inhibitors may offer modest reductions in Lp(a) alongside LDL-C lowering and were used here as part of a broader secondary prevention strategy. Dual antiplatelet therapy was initiated in accordance with current guidelines for non-cardioembolic stroke. This case illustrates the potential utility of incorporating Lp(a) measurement as part of the broader diagnostic and therapeutic assessment for young stroke patients with unexplained or multifactorial risk profiles.

## Lead author biography


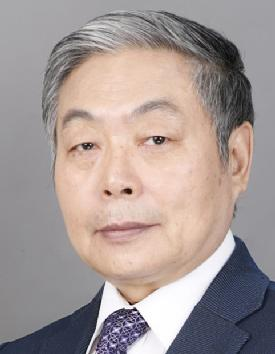


Dr Pingsheng Wu is a senior cardiologist specialising in hypertension, coronary artery disease, and heart failure. He works at East-West Medical Centre, Oka Medical Centre, and Nanfang Hospital, Southern Medical University, Guangzhou, China. Dr Wu graduated from First Military Medical University, Guangzhou, in 1978 and conducted research at Kanazawa University, Japan, in 1994 and 2000.

## Data Availability

The data underlying this article are available to use for all readers.

## Notes

**Consent:** The authors confirm that written consent for the submission and publication of this case report, including images and associated text, has been obtained from the patient in accordance with COPE guidance.

**Funding:** None declared.
